# Multilayer Fluorescent Immunoassay for Early and Sensitive Dengue Virus Detection Using Host and Viral Biomarkers

**DOI:** 10.1021/acs.bioconjchem.5c00153

**Published:** 2025-06-12

**Authors:** Andrew S. Browne, Jieqiong Fang, Amany Elsharkawy, Tianwei Jia, Evan Reboli, Ying Luo, Xiaolin Sheng, Mukesh Kumar, Suri S. Iyer

**Affiliations:** † Department of Chemistry, Center for Diagnostics and Therapeutics, 1373 Georgia State University, 788 Petit Science Center, Atlanta, Georgia 30302, United States; ‡ Department of Biology, Petit Science Center, 1373 Georgia State University, Atlanta, Georgia 30302, United States; § Department of Chemistry, 14710 University of Massachusetts Lowell, 520 Olney Science Center, 265 Riverside Street, Lowell, Massachusetts 01854, United States

## Abstract

Early detection and monitoring of dengue virus (DENV) infections are critical for effective disease management. A comprehensive approach combining viral and host biomarker detection improves diagnostic accuracy. Here, we describe a signal enhancement technique combining fluorescent silica nanoparticles and bioorthogonal chemistries for the ultrasensitive detection of monocyte chemoattractant protein 1 (MCP-1), interferon gamma-induced protein 10 (IP-10), and the viral biomarker nonstructural protein 1 (NS1). Our plate-based sandwich assay enhances signals with multiple layered fluorescent dye-encapsulated nanoparticles. In human serum, the assay achieved a limit of detection (LOD) of 43 pg/mL (∼5.0 nM) for MCP-1, ranging from 100 pg/mL to 100 ng/mL; 66 pg/mL (∼7.6 nM) for IP-10, ranging from 10 pg/mL to 100 ng/mL; and 351 pg/mL (8.6 nM) for NS1, ranging from 100 pg/mL to 10 μg/mL. We also monitored host biomarkers in dengue virus-infected AG129 mice using a Milliplex Mouse Cytokine/Chemokine Magnetic Bead Panel. MCP-1 levels in infected mice ranged from 1000 to 7000 pg/mL (mean: 2911 pg/mL), while uninfected controls showed much lower levels (1–10 pg/mL, mean 7 pg/mL). IP-10 levels ranged from 150 to 300 pg/mL (mean 188 pg/mL) in infected mice and 50–100 pg/mL (mean 69.4 pg/mL) in controls. These results aligned with our multilayered fluorescent assay, demonstrating its potential for sensitive dengue biomarker detection.

## Graphical abstract

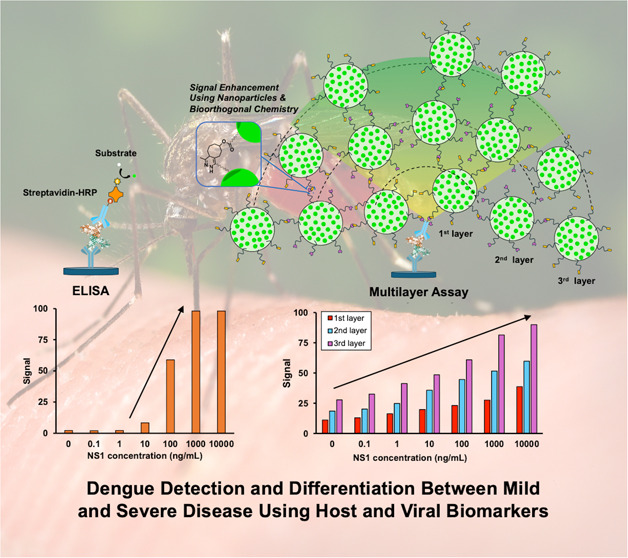

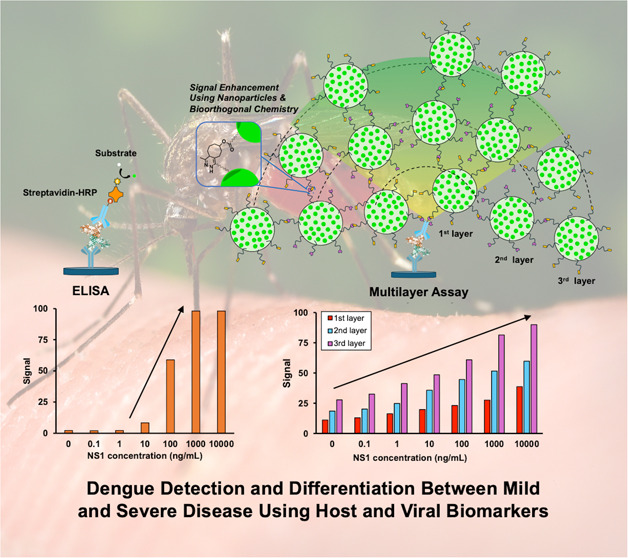

## Introduction

Dengue fever remains a major challenge to global health, in part due to the lack of specific antivirals and the limited availability of a vaccine that provides protection primarily against secondary infection.^1^ Early detection and monitoring of mosquito-borne viral infections are essential for effective clinical management and treatment. Dengue virus (DENV) belongs to the *Flaviviridae* family (genus *Flavivirus*) and comprises four antigenically distinct serotypes (DENV-1, DENV-2, DENV-3, and DENV-4).^2,3^ Infection with one serotype confers lifelong immunity to that serotype, yet subsequent infections with different serotypes can lead to severe disease manifestations such as dengue hemorrhagic fever (DHF) and dengue shock syndrome (DSS). is the primary vector, although also contributes to dengue transmission.^4–6^ Factors including urbanization and increased global travel have further expanded the geographic reach of dengue.^7,8^ This virus can present in varying clinical forms, ranging from classical dengue fever (DF) to severe dengue, including DHF (levels I and II) and DSS (levels III and IV).^9,10^ Viral proteins (for example, NS1) and host inflammatory mediators (for example, MCP-1 and IP-10) can compromise endothelial cell integrity and increase vascular permeability, a hallmark of severe dengue.^11–14^ Consequently, early detection and monitoring of dengue infection are crucial to preventing the progression to severe disease.^15–18^

The World Health Organization (WHO) estimates that there are hundreds of millions of dengue infections annually, with a significant portion manifesting clinically.^19,20^ The similarity of dengue symptoms to those of other febrile illnesses complicates diagnosis. Traditional approaches, such as virus isolation or nucleic acid detection via PCR, offer high specificity but are limited by their reliance on sophisticated laboratory infrastructure, higher cost, longer turnaround times, and the requirement for skilled personnel, making them less practical for rapid, point-of-care diagnostics.^19,10^ In contrast, immunoassays such as ELISA and rapid diagnostic tests (RDTs) that detect antidengue IgM/IgG antibodies or nonstructural protein 1 (NS1) antigen offer simpler, faster, and more scalable alternatives. These serological methods are particularly valuable in resource-limited settings and support timely clinical decision-making.^21–25^ Rapid diagnostic tests (RDTs) that detect nonstructural protein 1 (NS1) or IgM/IgG antibodies also support timely interventions.^19,10^ Despite these diagnostic tools, the complex pathophysiology of dengue underscores the need for enhanced diagnostics capable of identifying infections early and accurately tracking disease progression.

Dengue biomarkers, including NS1 and host inflammatory molecules such as MCP-1 and IP-10, can inform clinical management by indicating disease severity.^26^ Elevated MCP-1 and IP-10 levels, as well as other cytokines and chemokines (for example, IL-6, IL-8, and TNF-α), are associated with more severe cases, including DHF and DSS.^27^ NS1 also plays a critical role in early diagnosis.^28^ Elevated MCP-1 and IP-10 levels, as well as other cytokines and chemokines, are associated with more severe cases, including DHF and DSS.^27^ Previous studies of DENV patient serum have delineated the clinical concentration ranges of several host biomarkers. Table 1 compares the approximate serum or plasma concentrations of MCP-1, IP-10, and NS1 biomarkers in mild dengue fever and in severe disease (dengue hemorrhagic fever/severe dengue). These ranges are only estimates because concentrations vary with the day of illness, patient age, viral serotype, and whether the infection is primary or secondary. Taken together, integrating host biomarker with viral biomarker measurements provides comprehensive insights into dengue pathogenesis and progression, and this will help stratify patients according to their disease severity.^29^

**Table 1:** Concentrations of IP-10, MCP-1, and NS1 in Mild and Severe Dengue Infections

biomarker	mild dengue (DF)	severe dengue (DHF/DSS)	references
IP-10	elevated (∼300–500 pg/mL)	higher in early stages (∼500–700 pg/mL), declines in severe stages	^30–32^
MCP-1	elevated (∼200–400 pg/mL)	sustained elevation (∼400–600 pg/mL)	^33,34^
NS1	detectable (∼100–300 ng/mL)	higher levels (>500 ng/mL) associated with severe outcomes	^35,36^

Herein, we introduce a fluorescent, ultrasensitive multilayer sandwich immunoassay designed to quantify key host biomarkers, IP-10 and MCP-1, and the viral protein NS1. The assay employs bioorthogonal chemistry with fluorescent silica nanoparticles functionalized by methyltetrazine (Tz) and transcyclooctene (TCO).^37,38^ This chemistry allows rapid assembly of multiple layers of fluorescein isothiocyanate (FITC)-loaded silica nanoparticles, resulting in a substantially amplified signal.^39–41^ A schematic of the platform is depicted in Figure 1. The wide dynamic range of these multilayered assays covers the clinically relevant ranges from healthy individuals to patients with severe dengue infection. The assay also enables precise quantification of biomarker concentrations at various stages of infection, capturing a detailed picture of disease progression in AG129 mice infected with dengue virus serotype 2 (DENV-2).^42^ By tracking biomarker expression over time, the assay offers valuable insight into the pathogenesis of dengue and can alert clinicians to patients with increasing disease severity.^9,43–45^

**Figure 1:**
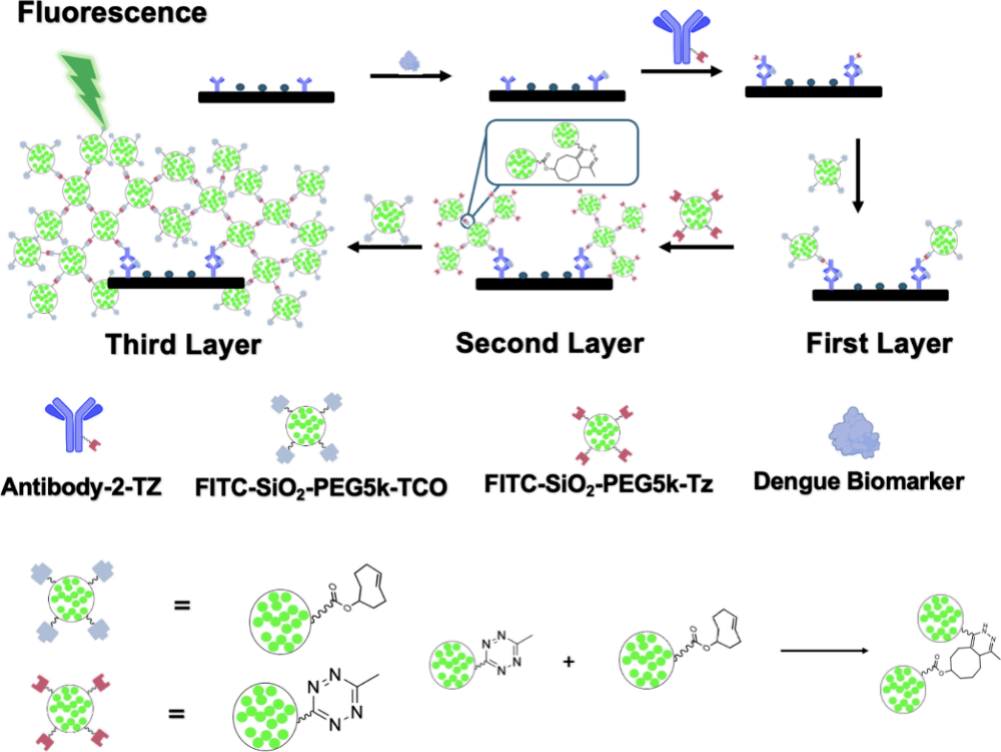
Schematic representation of the multiple layers used to enhance the signal for the ultrasensitive detection of dengue biomarkers.

## Experimental Section

### Materials and Equipment

Transcyclooctene-PEG6-NHS ester (NHS-PEG_6_-TCO), methyltetrazine-PEG5-NHS ester (NHS-PEG_5_-TZ), methyltetrazine-PEG4-amine HCl salt (NH_2_–PEG_4_-TZ), transcyclooctene-PEG6-amine (NH_2_–PEG_6_-TCO), and PEG-bis–CH2COOH (HOOC–PEG5k–COOH) were purchased from BroadPharm (San Diego, California). MCP-1 ELISA kits (human and mouse), recombinant MCP-1 antigen (human and mouse), and recombinant capture and detection MCP-1 antibodies (human and mouse) were purchased from Abcam. The NS1 ELISA kit was purchased from Eagle Biosciences. Viral Dengue Virus NS1 capture and detection antibodies, recombinant viral Dengue Virus 2 NS1 protein, human recombinant IP10 protein, and human IP10 antibody were purchased from R&D systems. Bovine serum albumin, mouse recombinant IP10 protein, mouse IP10 antibody, IP10 ELISA kit (human and mouse), black 96-well immuno plates (MaxiSorp), Cytiva Nanosep centrifugal devices with Omega membrane (30K, red), Pierce BCA protein assay kits, and normal mouse serum (Invitrogen) were purchased from Thermo Fisher Scientific. Ultrapure deionized water was obtained from a Millipore water purification system (18.2 MΩ cm^–1^, Milli-Q, Merck Millipore, Darmstadt, Germany). 200-proof ethanol was purchased from Decon Laboratories. Fluorescein isothiocyanate (FITC), (3-aminopropyl) triethoxysilane (APTES), tetraethyl orthosilicate (TEOS), *N*-hydroxysuccinimide (NHS), anhydrous sodium hydroxide (NaOH), triethylamine (TEA), Tween 20, and human serum type AB (male) from male AB plasma were purchased from Sigma-Aldrich. NH_4_OH (ammonium hydroxide, 28–30%) and anhydrous dimethyl sulfoxide (DMSO) were purchased from ARISTAR ACS, VWR Chemicals BDH. Phosphate-buffered saline was purchased from Corning. AG129 mice were purchased from Jackson Laboratory, and dengue virus stock was obtained from BEI Resources. Fluorescence intensity was detected by a Gen 5 Synergy LX Multimode reader (Agilent, Winooski, VT), and a green filter was used in the assays (BioTek Instruments, Inc.). Transmission electron microscopy (TEM) images were generated using a Philips CM12 transmission electron microscope.

### Fabrication of 100 nm FITC-SiO_2_–PEG5k-TCO Nanoparticles

FITC-SiO_2_–PEG5k-COOH was synthesized following a modified procedure adapted from methods developed in our group and detailed in the Supporting Information (Figure S1).

### Fabrication of 100 nm FITC-SiO_2_–PEG5k-TZ Nanoparticles

The procedure was identical to that for FITC-SiO_2_–PEG5k-TCO nanoparticles, except NH_2_–PEG_4_-TZ was used in place of NH_2_–PEG_6_-TCO.

### Preparation of the Tetrazine-Modified Anti-NS1/IP-10 Antibodies (Ab2-TZ)

An amount of 100 μL of detector antibody (Ab2, 50 μg) was added in a 1.5 mL microcentrifuge tube. A 25 mM NHS-PEG_5_-TZ solution was freshly prepared by dissolving 1.5 mg of NHS-PEG_5_-TZ in 75 μL of DMSO and 37.5 μL of PBS (pH 7.4 for NS1, or pH 8.5 for IP-10, adjusted by TEA), resulting in a final concentration of 1.8% DMSO. Subsequently, 14 μL of the NHS-PEG_5_-TZ solution was added to the antibody solution along with 386 μL of PBS (pH 7.4 for NS1, or pH 8.5 for IP-10, adjusted by TEA), ensuring that the final mixture contained 1.8% DMSO. The mixture was stirred at rt for 5 h or kept in a refrigerator (0–4 °*C*) overnight. After the reaction was completed, the product was diluted and purified using a Nanosep 30K filter by washing three times with deionized water. The concentration of Ab-TZ was determined using BCA assays, and the solution was stored at −20 °C.

### Preparation of the Tetrazine-Modified Anti-MCP1 Antibody (Ab2-TZ)

Twenty-five μg amount of anti-MCP1 detector antibody was added to 224 μL of PBS buffer (pH 8.7, adjusted by TEA) for a final antibody content of 10% antibody. A 9.5 mg/mL solution of NHS-PEG_5_-TZ was freshly prepared in 33% PBS (pH 7.4) and 67% DMSO solution. Then, 100 equiv of NHS-PEG_5_-TZ were added directly into the antibody solution and mixed for 5 h (4 °C). The antibody was purified through buffer exchange in a Nanosep 30 000 centrifugal tube and stored in PBS (pH 7.4). Protein concentration was determined using BCA assays, and the solution was stored at −20 °C.

### Coating Maxisorp Plates

To coat the capture antibodies (Ab1) on the plate, 200 μL of PBS (pH 8.0, adjusted with NaOH) was added to each well, and the samples were incubated for 30 min on a plate shaker to enhance electrostatic interactions. The liquid was carefully removed. Three μg of antibody was added to each well. The 96-well plate was incubated with the antibody for 24 h (4 °C).

### Blocking Plates

To block exposed surfaces on the plate and minimize nonspecific binding, the coating liquid was carefully removed, and the wells were washed twice with 200 μL of wash buffer (0.1% BSA and 0.05% Tween 20 solution). Subsequently, 300 μL of 0.1% BSA and 0.05% Tween 20 solution was added to each well, and the mixture was incubated at RT for 30 min on a rotor.

### Multilayer Assay for Biomarker Detection

A multilayered plate assay was employed, in which 10 μL of serum was diluted with 90 μL of wash buffer to reach the required volume for analysis. The assay design allowed for up to four separate analyses per sample, thereby maximizing the utility of the collected serum. The sensitivity range was calibrated with a standard curve spanning 1 pg/mL to 100 ng/mL of the analyte, appropriate for expected concentrations of biomarkers such as MCP-1. This setup enabled precise quantification of biomarker levels in the samples. Each assay was performed in duplicate or triplicate.

### ELISA and Validation Assays

To corroborate the findings from the multilayered assay, an ELISA protocol was implemented, adhering to a similar sample preparation methodology. The ELISA assay was complemented by a standard curve mirroring the concentrations used in the multilayered assay, ensuring consistency in quantification across different methodologies. The standard ELISA required 10 μL of sample and was further diluted with the respective kit’s dilution buffer following the kit’s protocol.

### Virus Stocks

DENV-2 NGC (NR-84) was obtained from BEI Resources. Virus stocks were prepared by infecting BHK21 cells with a multiplicity of infection of 0.01 in DMEM. Medium collected at day 5 post inoculation was clarified, aliquoted, and stored at −80 °C.

### In Vivo Dengue Infection Study

We performed the infectious DENV-2-related in vivo mouse experiments in the Animal Biosafety Level 2 laboratory (ABSL-2) in accordance with the recommendations in the Guide for the Care and Use of Laboratory Animals of the National Institutes of Health. The protocols were approved by the Institutional Animal Care and Use Committee at Georgia State University (Protocol number A24041). In these experiments, we used AG129 mice that were obtained from Jackson Laboratory (strain no. 029098). AG129 mice are C57BL/6 mice that are deficient in both IFNαβ and IFNγ receptors and are susceptible to severe disease after DENV infection. Six-week-old AG129 mice were inoculated intraperitoneally with 10^5^ PFU of DENV-2 or saline as mock.^25^ An equal number of male and female mice were used for each group. At day 5 post infection, mice were euthanized under isoflurane, and blood was collected via cardiac perfusion. Serum was separated for further analysis.

### Virus Titration in the Serum via Plaque Assay

Serum samples studied were sera collected from DENV-2- and mock-infected mice. The serum sample used in the assay was confirmed as representing positive for DENV-2 by a plaque assay. Briefly, BHK21 cells were seeded at a density of 2 × 10^5^ cells per well in six-well tissue culture plates for 3 days to form monolayers. To titrate the infectious virus in the serum sample, we serially diluted the serum 10-fold, starting at 1:10, in the cell infection medium and applied it to monolayered BHK21 cells for 1 h at 37 °C, with periodic rocking every 15 min. After inoculation, the cells were overlaid with 1% low-melting agarose. Five days after virus inoculation, the plates were stained with 2% neutral red in 1% low-melting agarose for visualizing plaque formation.^46^

### MCP-1 and IP-10 Protein Measurements

We analyzed serum samples collected at day 5 after infection with DENV-2 virus or saline (mock) for MCP-1 and IP-10 levels using the Milliplex Mouse Cytokine/Chemokine Magnetic Bead Panel (Millipore Sigma, Cat# MCYTMAG70PMX25BK). Sample concentrations were calculated using the Belysa Immunoassay Curve Fitting Software (Millipore Sigma).^26^

### Statistical Analysis

All experiments were performed with at least two independent replicates. Data are presented as mean ± SEM (error bar). Statistical significance was assessed using unpaired, two-tailed Student’s *t*-tests, with *p* < 0.05 considered statistically significant.

### Safety Statement

This study involved hazardous chemicals (e.g., DMSO, ammonium hydroxide, sodium hydroxide, and ethanol), which were handled using standard PPE (lab coats, gloves, and goggles) and in a fume hood. Biological materials, including dengue virus proteins and human/mouse serum, were handled under Biosafety Level 2 (BSL-2) conditions, and animal work followed the approved IACUC protocols. Nanomaterials such as PEGylated compounds and silica nanoparticles were managed by using appropriate ventilation and safety practices. No unexpected or unusually high hazards were encountered.

## Results and Discussion

All materials used in our assays were synthesized and fully characterized, as shown in Figure S1 (synthetic scheme), Figure 2 (TEM images), and Figure S2 (zeta potential analysis). FITC dye-doped fluorescent nanoparticles were used, with surface modifications of either tetrazine (TZ) or transcyclooctene (TCO), resulting in the formulations FITC-SiO_2_–PEG5k-TCO and FITC-SiO_2_–PEG5k-TZ, respectively. The nanoparticles, approximately 100 nm in diameter, were prepared using previously established methods, with the detailed procedure provided in Figure S1.^39^ Surface modifications were achieved through the use of a polyethylene glycol spacer (HOOC-PEG5k-COOH, molecular weight 5000 g/mol) terminated with a carboxyl group, to which TCO or TZ was subsequently conjugated. The spherical morphology of the nanoparticles, with an average diameter of ∼100 nm, was confirmed through transmission electron microscopy (TEM) imaging, as shown in Figure 2. Zeta potential measurements were conducted, yielding values of −5.16 ± 0.08 mV for FITC-SiO_2_–PEG5k-TCO and −6.80 ± 1.26 mV for FITC-SiO_2_–PEG5k-TZ, as displayed in Figure S2. This characterization demonstrates the successful synthesis and surface modification of the nanoparticles employed in the assays.

**Figure 2:**
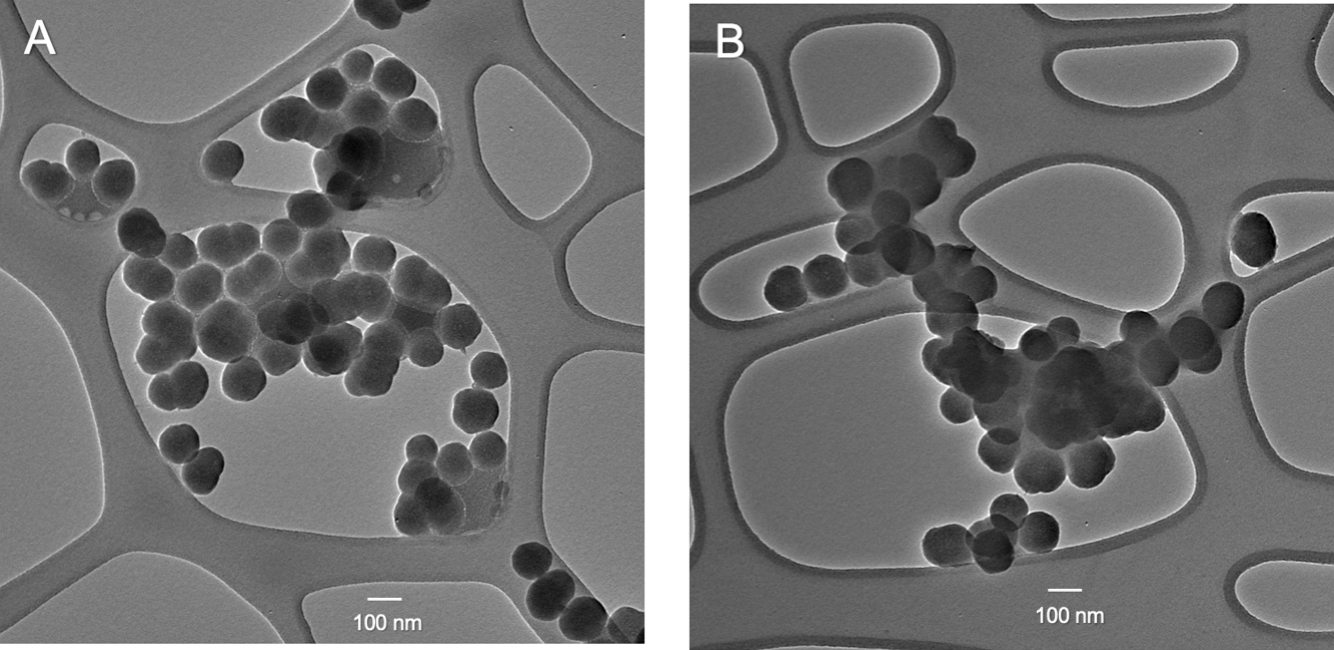
TEM images of fluorescent silica nanoparticles. (A) FITC-SiO_2_–PEG5k-TZ. (B) FITC-SiO_2_–PEG5k-TCO.

### Feasibility Studies

To prove the concept of the multilayered fluorescent assay, a feasibility study was performed on 96-well plates. For MCP-1, the assay was performed with an experimental group of 1 μg/mL MCP-1 and a control group that contained only phosphate-buffered saline (PBS). A higher fluorescent signal was observed from the first layer in the experimental group compared with the control group, confirming that FITC-SiO_2_–PEG5k-TCO was successfully conjugated to Ab-TZ via the TCO-TZ bioorthogonal reaction. In the second layer, an increased signal intensity was detected relative to the first layer’s experimental group, whereas the signal in the control group remained relatively unchanged from the first to the second layer. This observation indicates that unreacted TCO groups interacted with the paired FITC-SiO_2_–PEG5k-TZ, leading to the formation of additional layers. In the third layer, a considerably higher signal was produced compared with the control group. A larger separation of signal to noise takes place with each additional layer of nanoparticles, effectively enhancing the signal. This signal enhancement can be seen in Figure 3. The feasibility study results for NS1 and IP-10 are presented in Figures S4 and S5.

**Figure 3:**
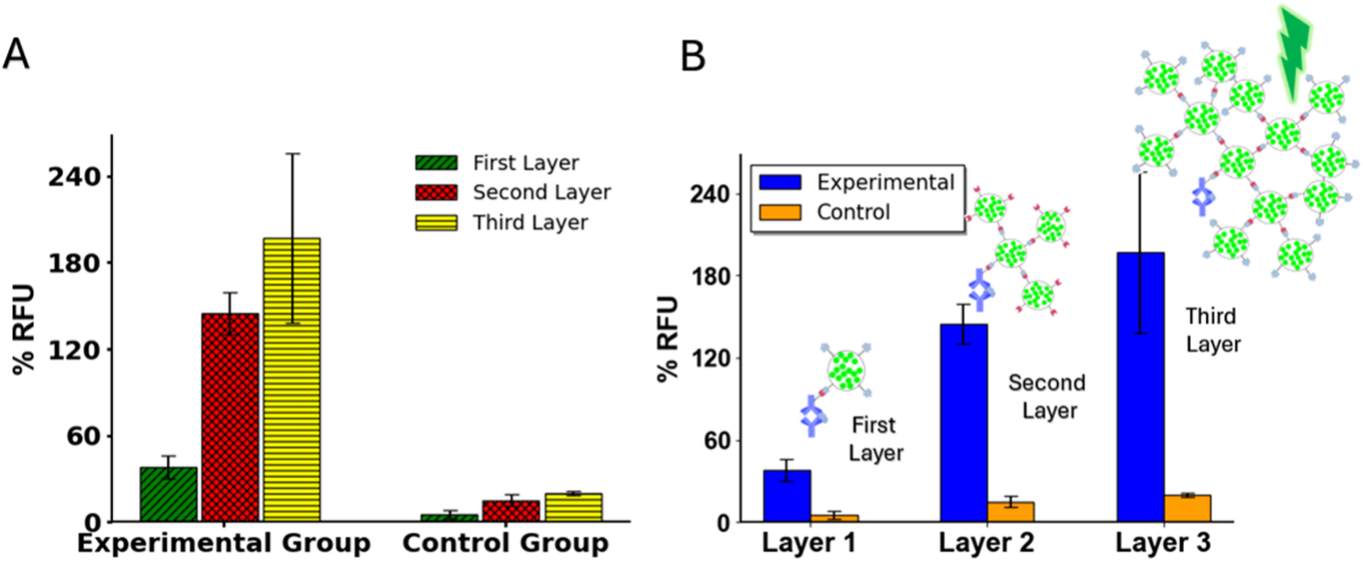
Demonstration of the signal enhancement strategy using the MCP-1 antigen. The *y*-axis, % RFU, represents the percent relative fluorescence intensity of the sample as a function of external reference signal of 100 nM fluorescein. (A) % RFU functions organized by the experimental group and the control group. (B) % RFU values organized by layers, illustrating the incremental signal enhancement achieved with each additional layer.

We further optimized the assay by evaluating: (1) different compositions of blocking reagents, (2) various Ab-TZ conjugation conditions, including PBS buffer pH, temperature, and reaction time, and (3) a reduced overall incubation time. We observed that decreasing the BSA concentration in the blocking solution from 1% to 0.1% significantly reduced the background signal (Figure S3). For NS1 detection, Ab-TZ conjugation at pH 7.4, rt, 5h, resulted in a substantial signal increase compared to the control. In contrast, for IP-10 detection, pH 8.5, 0 °C, overnight conjugation conditions yielded better results. To explore the feasibility of adapting the multilayered assay for point-of-care testing (POCT), we reduced the total incubation time from 75 to 25 min. While the fluorescence signal intensity decreased significantly, the experimental-to-control (E/C) ratio remained comparable, indicating the potential for rapid detection. The results are presented in Figures S4 (for NS1 feasibility and optimization studies) and S5 (for IP-10 feasibility and optimization studies), with E/C values summarized in Tables S1 (for NS1) and S2 (for IP-10).

### Limit of Detection Studies

To determine assay sensitivity, varying concentrations of NS1, MCP-1, and IP-10 were tested to generate standard curves of signal versus concentration. As shown in Figure 4, the fluorescent multilayer immunoassay allowed for sensitive detection of these dengue-associated biomarkers in PBS. The *y*-axis (RFU%) reflects fluorescence signals normalized to standard fluorescein solutions (40 nM for IP-10 and NS1, and 100 nM for MCP-1). A linear regression model was produced for each dengue biomarker observed; for example, the linear regression model for the first layer of MCP-1 was *y* = 16.57*x* + 5.02, *R* ^2^ = 0.97, where *x* is the concentration of the MCP-1 antigen, and *y* is the % RFU. The value of the LOD is calculated using the formula: LOD = mean blank signal +3σ, where σ represents the value of the standard deviation of blank samples. According to the formulas, the assay reached detection limits of 4 pg/mL for MCP-1, 159 pg/mL for IP-10, and 205 pg/mL NS1, as evidenced by significant increases in %RFU over background (*p* < 0.05). The assay showed good reproducibility. Intra-assay variation for IP-10 detection was low, with an average CV of 2.15% (Figure S3). Interassay CVs averaged 12.24% across 3 days and decreased with each amplification layer for both IP-10 and NS1, as shown in Figure S4 and summarized in Table S5. Collectively, these results underscore the high analytical sensitivity of the multilayer immunoassay, underscoring its potential for early and precise detection of dengue biomarkers.

**Figure 4:**
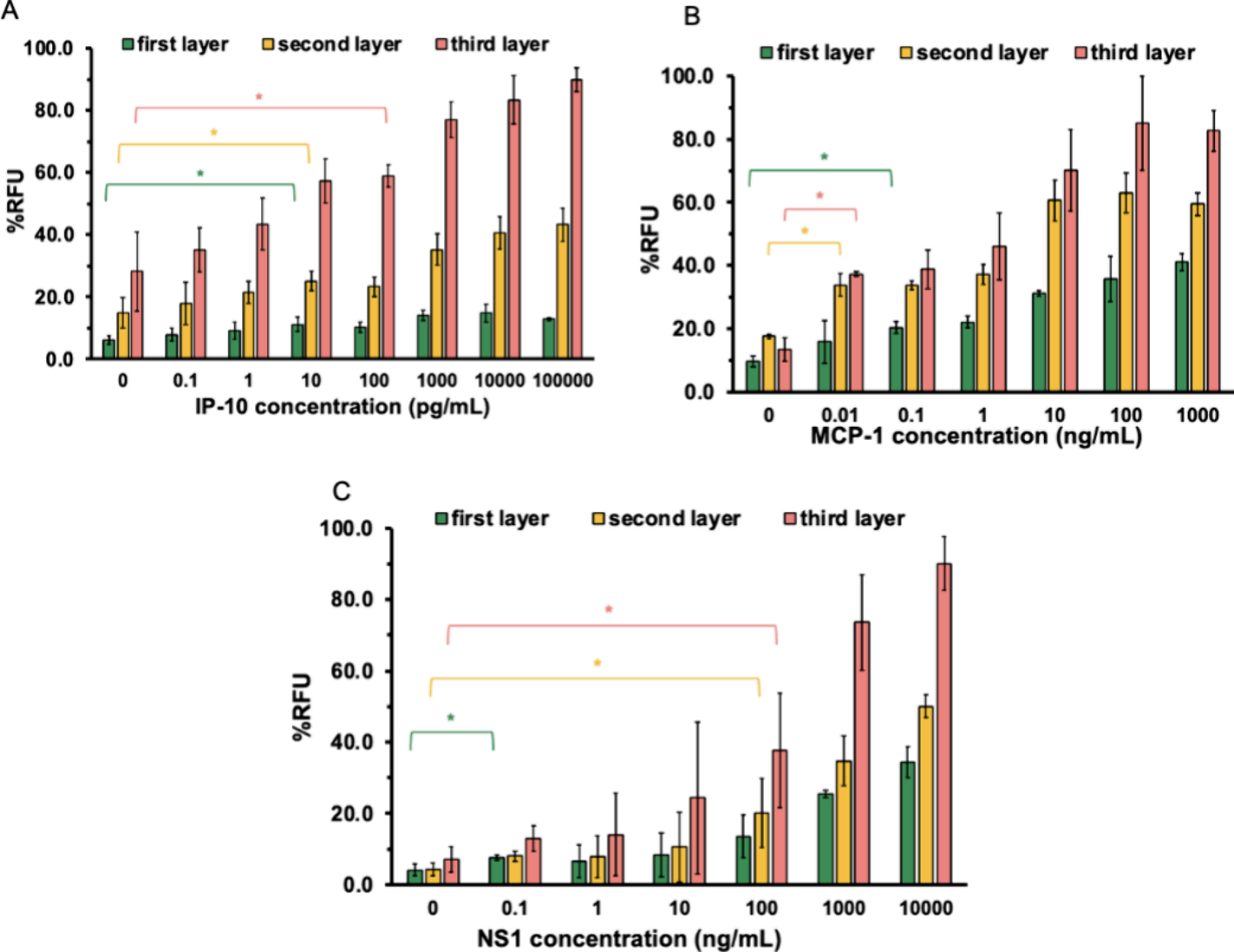
Signal response of fluorescent multilayer immunoassay for dengue prognostic markers in PBS: limit of detection of (A) IP-10, (B) MCP-1, and (C) NS1. The *y*-axis, RFU%, represents the fluorescence intensity of the sample divided by fluorescein standard solution for the biomarkers. Error bars indicate standard deviations of three measurements for the biomarkers IP-10 and NS1. Error bars represent experiments done in duplicate for the biomarker MCP-1 (ns >0.05, * *p* < 0.05, ** *p* < 0.01, ***p < 0.001).

To evaluate assay performance in a biologically relevant matrix, we determined the limit of detection (LOD) in human serum using a procedure analogous to that used for buffered samples. Using serum samples demonstrates assay specificity, as serum contains a significant number of biomolecules that could potentially interfere with the binding event. Varying concentrations of NS1, MCP-1, and IP-10 were tested to generate standard curves of the signal versus concentration. As shown in Figure 5A–C, the fluorescent multilayer immunoassay allowed for sensitive detection of these dengue-associated biomarkers in serially diluted serum. The *y*-axis (RFU%) reflects fluorescence signals normalized to standard fluorescein solutions (40 nM for IP-10 and NS1, and 100 nM for MCP-1). The assay reached detection limits of 43 pg/mL for MCP-1, 66 pg/mL for IP-10, and 351 pg/mL NS1, demonstrating significant increases in RFU% over background (*p* < 0.05). Error bars indicate triplicate measurements for IP-10 and NS1, and duplicate measurements for MCP-1**.** If the assays were heavily influenced by nonspecific binding, we would expect to observe alterations in the linearity of the assay signal response from the serum experiments and also increased standard deviation error bars in comparison to the experiments in buffer. However, our assays retained strong performance in serum with minimal background interference, and the signal remained linear, closely matching the curve obtained in buffer for each biomarker. These results confirm that the multilayer immunoassay maintains high analytical sensitivity in complex biological matrices, highlighting its potential for clinical diagnostics.

**Figure 5:**
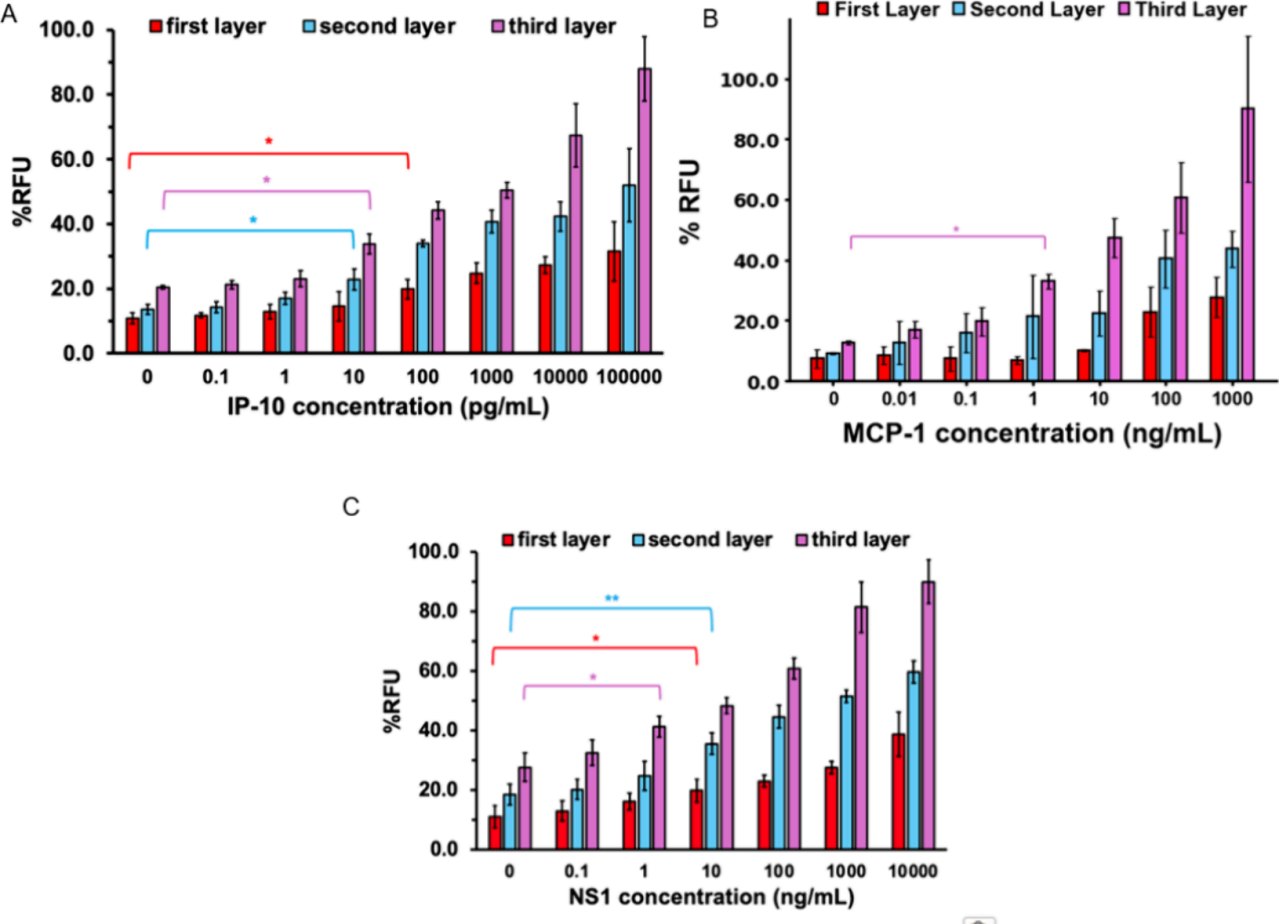
Signal response of fluorescent multilayer immunoassay for dengue prognostic markers in diluted mouse serum: limit of detection of (A) IP-10, (B) MCP-1, and (C) NS1. The *y*-axis, RFU%, represents the fluorescence intensity of the sample divided by fluorescein standard solution for the biomarkers. Error bars indicate standard deviations of three measurements for the biomarkers IP-10 and NS1 (A,B). Error bars represent experiments done in duplicate for MCP-1 (C) (ns >0.05, * *p* < 0.05, ** *p* < 0.01, ***p < 0.001).

### Detection of Dengue Biomarkers in AG129 Mice Samples

The multilayered fluorescent assay was employed to measure these biomarkers in the serum samples collected at day 5 after the infection with DENV-2, in parallel with standard ELISA kits, allowing for an apples-to-apples comparison of the two methods.

Using a multiplex immunoassay assay, we quantified the protein levels of MCP-1 and IP-10 for reference in the serum samples collected 5 days after the infection with DENV-2. Compared with mock-infected serum samples (healthy samples), we detected significantly higher levels of MCP-1 in DENV-2-infected serum samples (*p* value= 0.0154). The mean protein concentration of MCP-1 was 2,911 pg/mL in the DENV-2 infected serum samples and 7.2 pg/mL in the mock-infected serum samples (Figure 6A). Additionally, we detected a significant increase in the protein levels of IP-10 in DENV-2-infected serum (*p* value= 0.0005). The mean concentration of IP-10 was 188 pg/mL in the DENV-2-infected serum samples and 69.4 pg/mL in the mock-infected serum (Figure 6B). Serum samples were confirmed as representing positive DENV-2 infection by the plaque assay. The mean virus titer in the DENV-2-infected serum sample was 7 × 10^4^ PFU/mL (Figure 6C). Notably, high levels of viremia correlated to the increased levels of MCP-1 and IP-10 in the serum of DENV2-infected mice (Figure 6).

**Figure 6:**
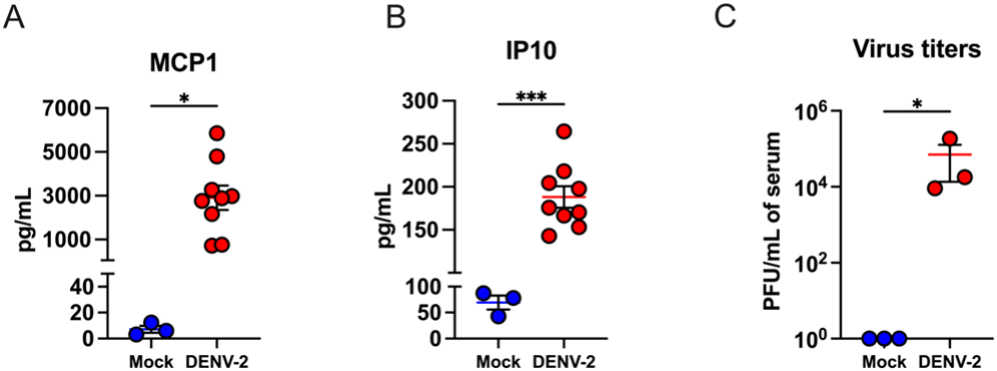
MCP-1 and IP-10 protein levels in diluted mouse serum. Serum samples were collected from mock- and DENV-2-infected animals at day 5 after the infection. (A) Protein levels of MCP-1 in the serum of mock- and DENV-2-infected mice. (B) Protein levels of IP-10 in the serum of mock- and DENV-2-infected mice. The middle bar indicates the mean ± SEM. Each point represents an individual mouse (*n* = 3, 9 mice for mock- and DENV2-infected serum samples, respectively). Statistical significance was determined by the unpaired *t* test (*p < 0.05; ** *p* < 0.01; *** *p* < 0.001, **** *p* < 0.0001). (C) Infectious virus titers were determined in the serum of mock- and DENV-2-infected mice via plaque assay (*n* = 3 per group). Statistical significance was determined by the Mann–Whitney U test (*p < 0.05; ** *p* < 0.01; *** *p* < 0.001, **** *p* < 0.0001).

The analyte concentrations in mouse serum samples collected 5 days postinfection with DENV-2 and from mock (saline-treated) controls are summarized in Table 2. The multilayered fluorescent nanoparticle signal enhancement assay was employed to detect dengue prognostic biomarkers, including the viral protein NS1 (Figure S6) and the nonviral proteins IP-10 (Figure S7) and MCP-1 (Figure S8). For comparison, conventional ELISA assays were conducted in parallel, with corresponding results also shown in Figures S6–S8. Compared with ELISA, our assay enabled precise quantification and enhanced sensitivity in detecting cytokines and chemokines, providing a robust analysis of differences between infected and mock samples.

**Table 2:** Concentrations of Dengue Biomarkers in the AG129 Mouse Serum Using the Multilayered Fluorescent Assay

biomarker	concentration in DENV-2 infected serum sample (pg/mL)	concentration in mock-infected serum sample (pg/mL)
MCP-1	3162	794
IP10	1982	127
NS1	2.7 ·10^6^	0

## Conclusions

We developed a highly sensitive sandwich immunoassay that integrates fluorescent silica nanoparticles and bioorthogonal chemistries for the ultrasensitive detection of host biomarkers (MCP-1 and IP-10) and a viral biomarker (NS1) for early dengue diagnosis and monitoring disease progression. This approach uses a layer-by-layer approach using fluorescent nanoparticles and bioorthogonal chemistries. For the host biomarkers, the assay achieved LODs as low as 4 pg/mL for MCP-1 and 159 pg/mL for IP-10 in phosphate-buffered saline (PBS), and 43 and 66 pg/mL in serially diluted serum. For the viral biomarker NS1, LODs of 205 pg/mL in PBS and 351 pg/mL in serum were observed, demonstrating robust performance over a broad biomarker concentration range. The detection range was 100 pg/mL to 100 ng/mL for MCP-1, 10 pg/mL to 100 ng/mL for IP-10, and 100 pg/mL to 10 μg/mL for NS1. The multilayered fluorescent nanoparticle assay was used to monitor the concentrations of these biomarkers in infected AG129 mouse samples and mock-infected mouse samples, in parallel with ELISA tests. The assay was able to differentiate healthy and infected mouse samples.

We compared the potential value of this assay with existing methods that include ELISA, RDT, and RT-PCR for NS1 (Table 3).

**Table 3:** Comparison of the New Assay to ELISA, RDT and rt-PCR

attribute	this assay	NS1 ELISA (PanBio)	RDT (SD Dengue Duo)	Multiplex qRT-PCR
analytical LOD (NS1)	0.1 ng/mL, dynamic range from 100 pg/mL to 10 μg/mL	0.5 ng/mL; clinical sensitivity, 60–75%, Specificity 71–80%^47^	0.25–2 ng/mL^48,49^	genome-copy level^50^
clinical sensitivity and specificity	in progress	sensitivity, 60–75%; specificity 71–80%^47^	field sensitivity, 62–84%, field specificity 98–100% ^41,42^	clinical Specificity and selectivity >98%^50^
hands-on time and instrument	30 min; palm-sized fluorimeter	2–3 h; plate washer/reader	15 min; no reader	3–4 h; thermocycler + extraction
estimated per-test consumable cost	<$4 (materials only)	US $8–15 kit, cost is lower when purchased in bulk.	US $3–6,^51^ but cost in southeast Asia is much lower ∼$1.0	US $2.6 (rxn) + US $4 extraction,^52^ cost per test can be lower
ease-of-use in field	lab only, but efforts are underway to translate the assay to POC testing	lab only	self-testable	skilled operator
ability to test for viral and host biomarkers	yes	no	no	no

As shown in the table, the new assay offers significant advantages. Most importantly, current assays do not enable monitoring of disease severity. By measurement of viral (NS1) and host markers (MCP-1 and IP-10), this assay provides a complete picture of disease progression. Early detection of NS1 is critical for identifying dengue infection, while monitoring host biomarker levels helps guide timely clinical decisions to prevent severe outcomes such as hemorrhagic fever or shock.^8^

## Supplementary Material



## Notes

Fluorescent silica nanoparticles fabrication and characterization; optimization studies; comparison between multilayer assays and ELISA for AG129mice samples; and reproducibility (PDF)
