# Supplementary material for: Multilayer Fluorescent Immunoassay for Early and Sensitive Dengue Virus Detection Using Host and Viral Biomarkers
Source: Bioconjug Chem. 2025 Jun 12;36(7):1474–82. doi: 10.1021/acs.bioconjchem.5c00153 (PMC12272551; doi:10.1021/acs.bioconjchem.5c00153)
Supplement: Supplementary file 1 [file bc5c00153_si_001.pdf]

Supporting Information For Publication

Supporting Information

A Multilayer Fluorescent Immunoassay for Early and Sensitive Dengue Virus Detection using Host and Viral Biomarkers

Andrew S. Browne[1†], Jieqiong Fang[1†], Amany Elsharkawy[2], Tianwei Jia[1], Evan Reboli[3], Ying Luo[1], Xiaolin Sheng[1], Mukesh Kumar [2*] and Suri S. Iyer[1,3*]

[1] Georgia State University, Department of Chemistry, Center for Diagnostics and Therapeutics, 788 Petit Science Center, Atlanta, GA 30302, USA.

[2] Georgia State University, Department of Biology, Petit Science Center, Atlanta, GA 30302, USA.

[3] University of Massachusetts Lowell, Department of Chemistry, 1 University Avenue, Lowell, MA 01854, USA.

† These authors contributed equally to this work.

Email: suri_iyer@uml.edu; mkumar8@gsu.edu

Table of Contents

1. Fluorescent Silica Nanoparticles Fabrication and Characterization **1-3**

2. Optimization Studies **4-8**

3. Comparison between Multilayer Assays and ELISA for AG129 mice samples **9-11**

4. Reproducibility **12-14**

1. Fluorescent Silica Nanoparticles Fabrication and Characterization

1.1 Fluorescent Silica Nanoparticles Fabrication

All reactions were carried out in oven-dried glassware under nitrogen atmosphere, unless stated otherwise. Chemicals were purchased from Sigma-Aldrich, Fisher Scientific, Alfa Aesar or Acros and used without further purification. Anhydrous solvents were used without further treatment and distillation.

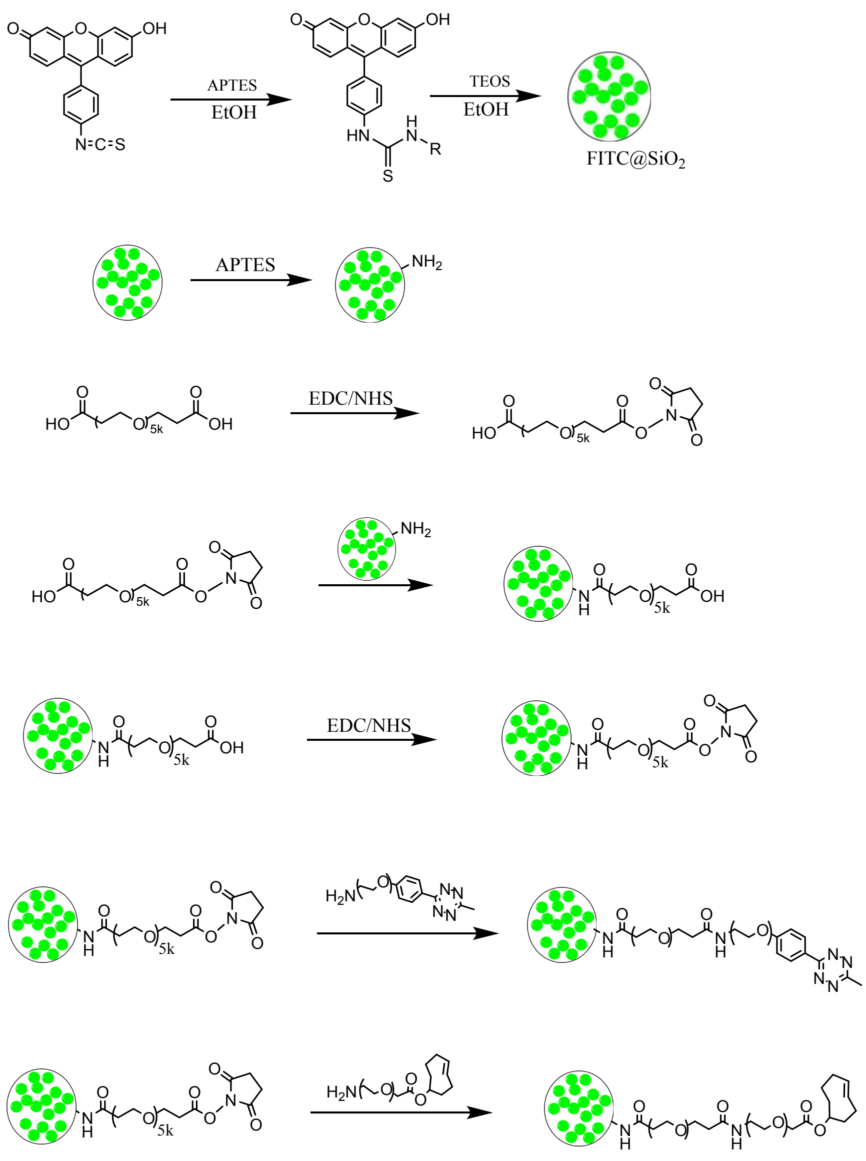

Figure S1. Scheme of Synthesis of Fluorescent Silica Nanoparticles.

Synthesis of 100 nm FITC-SiO$_2$-OH

FITC-SiO_2-OH nanoparticles were synthesized using a modified protocol based on established methods. FITC (10 mg), APTES (20 µL), and absolute ethanol (5 mL) were combined in a 50 mL round-bottom flask sealed with a rubber stopper. The mixture was stirred at room temperature for 20 hours to form the FITC-APTES adduct. Without altering the reaction conditions, ethanol (20 mL), TEOS (2.0 mL), NH_4OH (0.65 mL), and Milli-Q water (1.3 mL) were sequentially added, and the reaction proceeded for an additional 24 hours. The resulting yellow precipitate was collected and washed thoroughly with ethanol over 10 cycles (~25 mL per cycle), involving centrifugation (7000 rpm, 15 min), sonication, and redispersion. The purified product was redispersed in 10 mL of ethanol for storage.

Synthesis of FITC-SiO_2-NH_2

FITC-SiO_2-OH (60 mg) was suspended in ethanol (10 mL), degassed for 30 minutes, and sonicated for 15 minutes. APTES (400 µL) was added dropwise, and the reaction mixture was refluxed at 95 °C with continuous stirring for 36 hours. The resulting FITC-SiO_2-NH_2 nanoparticles were isolated by centrifugation (10,000 rpm, 15 min), washed three times with ethanol, and vacuum-dried for 2 hours.

Synthesis of FITC-SiO_2-PEG5k-COOH

HOOC-PEG5k-COOH (55 mg, 11 µmol) was dissolved in DMF (2 mL) under a nitrogen atmosphere. NHS (1.2 mg, 10 µmol) and EDC·HCl (1.9 mg, 10 µmol), each dissolved in 0.2 mL of DMF, were added dropwise to the solution and stirred for 30 minutes to activate the carboxyl groups. Meanwhile, FITC-SiO_2-NH_2 (30 mg) was dispersed in DMF (1 mL) and sonicated for 15 minutes before being added to the reaction mixture. The reaction was allowed to proceed under stirring for 20 hours. The product was purified by three rounds of centrifugation (10,000 rpm, 15 min) and washing with DMF. The final product was redispersed in DMF at a concentration of 10 mg/mL.

Fabrication of 100 nm FITC-SiO_2-PEG5k-TCO Nanoparticles

FITC-SiO_2-PEG5k-COOH (5 mg) was dispersed in DMF (1 mL) under a nitrogen atmosphere. NHS (1.2 mg) and EDC·HCl (1.9 mg), each dissolved in 200 µL of DMF, were added to the solution and stirred for 1 hour to activate the carboxyl groups. NH_2-PEG6-TCO (2 mg in 200 µL DMF) was then added, and the reaction continued for 24 hours. The product was purified by three

rounds of centrifugation (10,000 rpm, 10 min) and washing with ethanol and PBS. The final nanoparticles were redispersed in PBS at a concentration of 5 mg/mL and stored at 4 °C.

Fabrication of 100 nm FITC-SiO_2-PEG5k-Tz Nanoparticles

The synthesis procedure for FITC-SiO_2-PEG5k-TZ nanoparticles was identical to that of FITC-SiO_2-PEG5k-TCO, except that TZ-PEG4-NH_2 was used in place of NH_2-PEG6-TCO.

1.2 Zeta Potential of Fluorescent Silica Nanoparticles

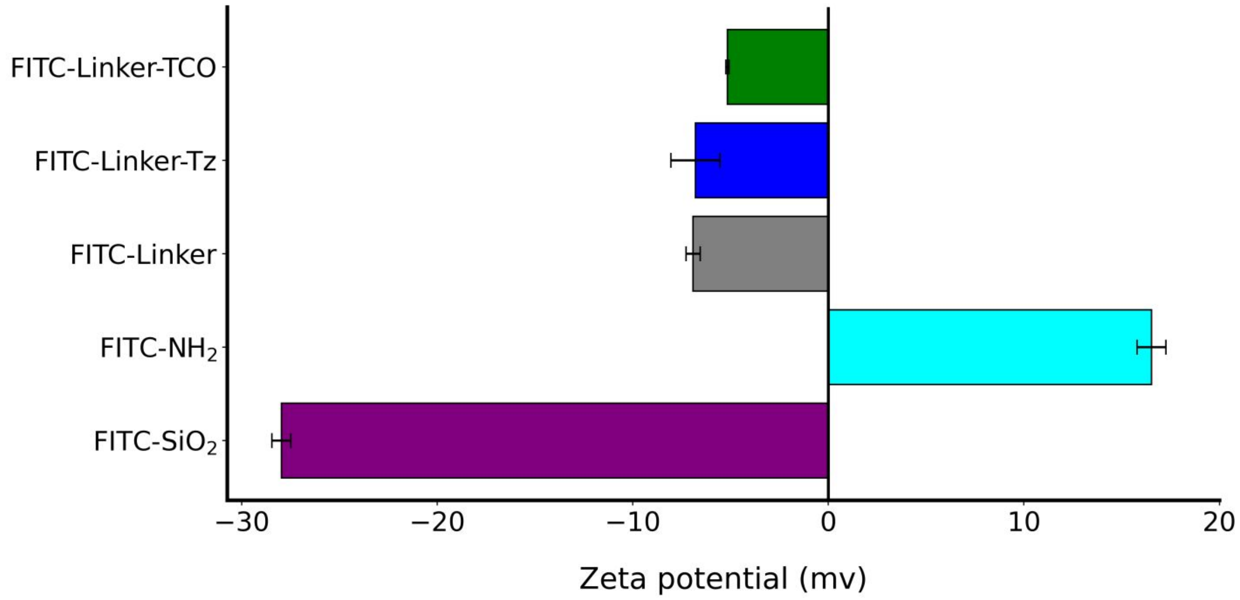

Figure S2. Zeta potential of fluorescent silica nanoparticles. Changes in the zeta potential are greatly correlated with changes in the nanoparticle's surface charge indicating successful alteration of the surface of the nanoparticles following each step of synthesis.

2. Optimization Studies

2.1 Blocking Agents

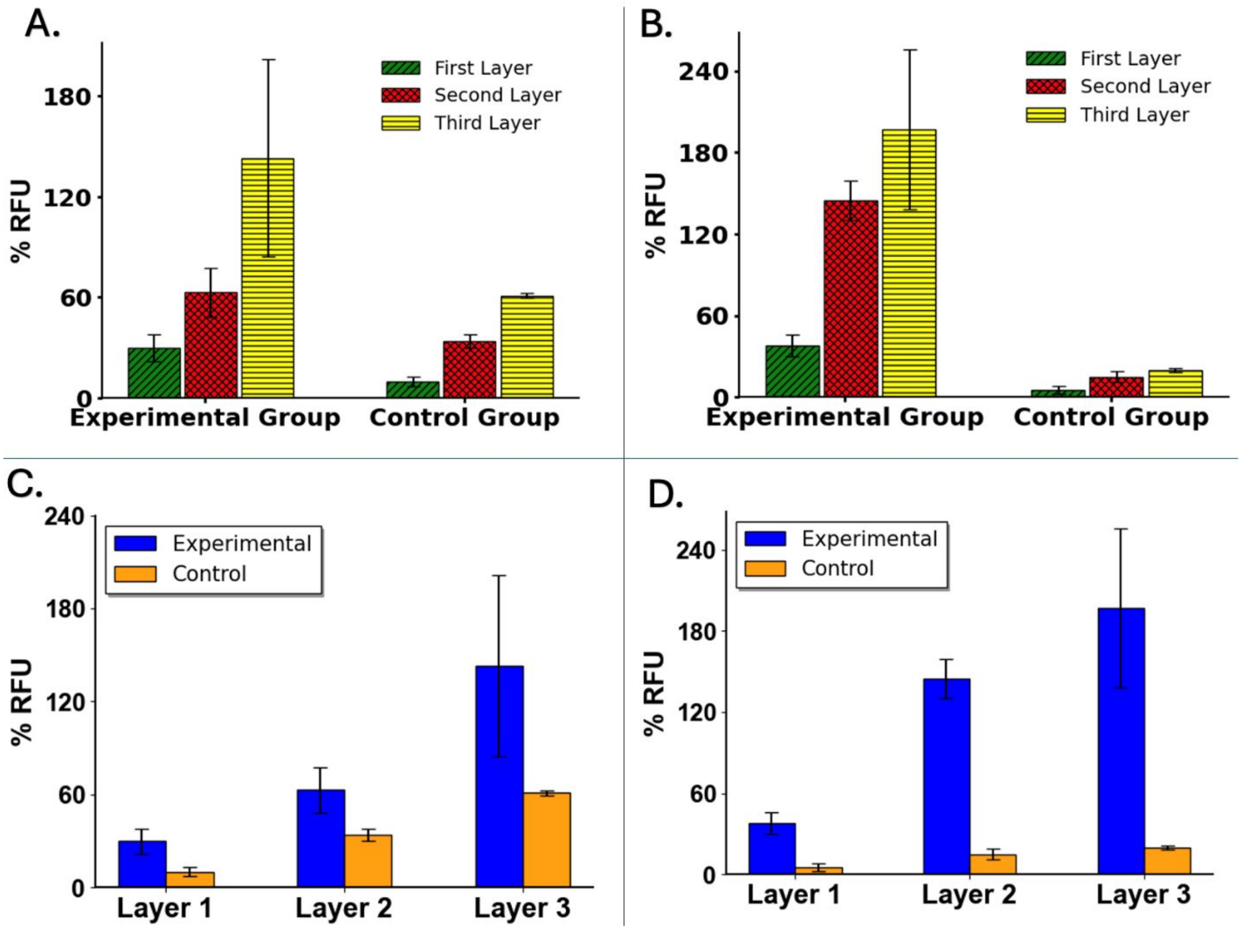

Figure S3 Feasibility and optimization studies of the plate-based multilayer assay for MCP-1 detection. (A) Assay performed after blocking 96 well plate with 1% BSA solution. (B) Layer by layer comparison of assay blocked with 1% (C) Assay performed after blocking 96 well plate with 0.1% BSA solution. (D) Layer by layer comparison of assay blocked with 0.1%. Decreasing the amount of BSA used in blocking solution provided a pronounced decrease in background signal. Error bars indicate standard deviations from two independent measurements performed on different days.

2.2 Antibody-Tetrazine (Ab-Tz) Conjugation Conditions and Incubation Time

For NS1

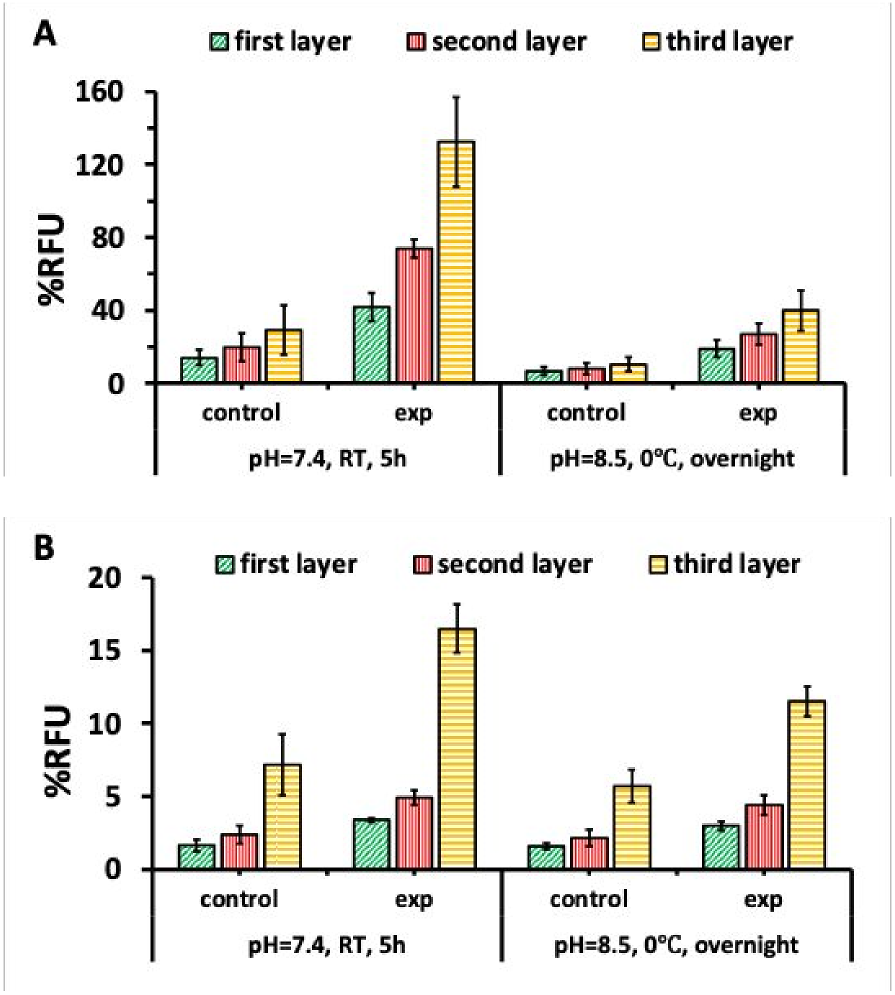

Figure S4 Feasibility and optimization studies of the plate-based multilayer assay for NS1 detection. (A) Assay with a total incubation time of 75 minutes, using Ab-Tz conjugation at pH 7.4, room temperature (RT), for 5 hours (left) or pH 8.5, 0°C, overnight (right). (B) Assay with a total incubation time of 25 minutes, using Ab-Tz conjugation at pH 7.4, room temperature (RT), for 5 hours (left) or pH 8.5, 0°C, overnight (right). The y-axis (%RFU) represents the fluorescence intensity of the sample normalized to a 40 nM fluorescein solution. Error bars indicate standard deviations from three independent measurements performed on different days.

Table S1 Experiment-to-control ratio (E/C) for NS1 detection using different incubation time and Ab-Tz conjugation conditions.

Ab-Tz	Time to Results	E/C		
		L1	L2	L3
pH=7.4, RT, 5 h	75min	2.99	3.75	4.53
pH=8.5, 0℃, overnight	75min	2.88	3.38	3.82
pH=7.4, RT, 5 h	25min	2.05	2.08	2.30
pH=8.5, 0℃, overnight	25min	1.87	2.05	2.01

For IP-10

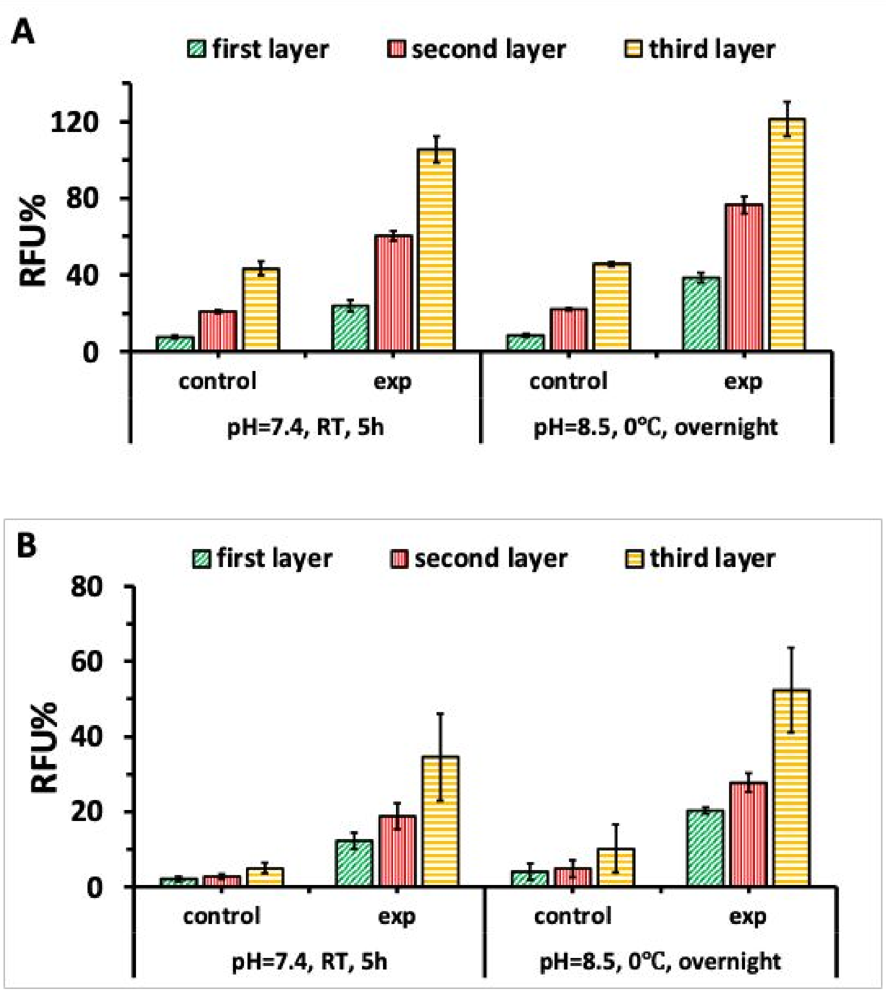

Figure S5 Feasibility and optimization studies of the plate-based multilayer assay for IP10 detection. (A) Assay with a total incubation time of 75 minutes, using Ab-Tz conjugation at pH 7.4, room temperature (RT), for 5 hours (left) or pH 8.5, 0°C, overnight (right). (B) Assay with a total incubation time of 25 minutes, using Ab-Tz conjugation at pH 7.4, room temperature (RT), for 5 hours (left) or pH 8.5, 0°C, overnight (right). The y-axis (%RFU) represents the fluorescence intensity of the sample normalized to a 40 nM fluorescein solution. Error bars indicate standard deviations from three independent measurements performed on different days.

Table S2 Experiment-to-control ratio (E/C) for IP10 detection using different incubation time and Ab-Tz conjugation conditions.

Ab-Tz	Time to Results	E/C		
		L1	L2	L3
pH=7.4, RT, 5 h	75min	3.19	2.89	2.43
pH=8.5, 0℃, overnight	75min	4.58	3.46	2.66
pH=7.4, RT, 5 h	25min	5.90	6.68	6.90
pH=8.5, 0℃, overnight	25min	4.98	5.67	5.14

3. Comparison between Multilayer Assays and ELISA for AG129 mice samples

For NS1

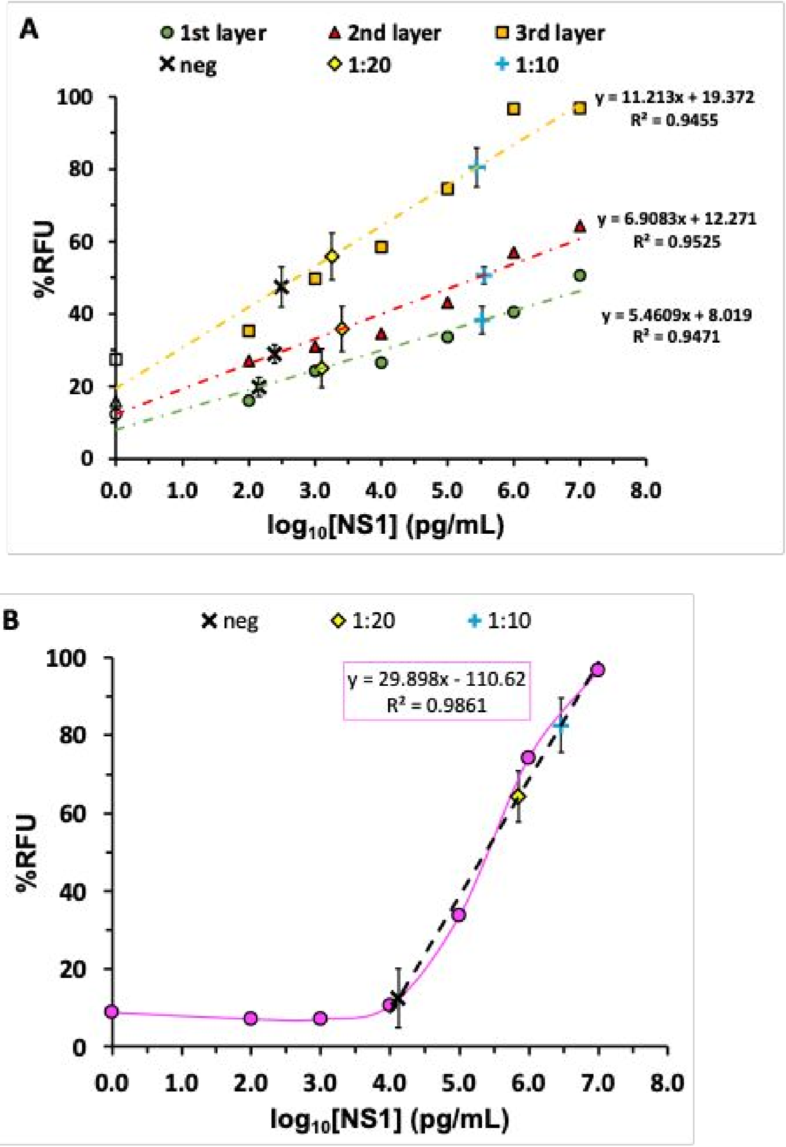

Figure S6 Comparison of the Multilayered Fluorescent Assay and Standard ELISA for detecting NS1 concentrations in mouse serum samples collected five days post-infection with the DENV2 virus and from mock (saline-treated) controls. (A) Detection using the Multilayered Fluorescent Assay. (B) Detection using the Standard ELISA with an ultrared fluorescent substrate. The infected samples were diluted 10-fold or 20-fold. The y-axis (%RFU) represents the fluorescence intensity of the sample normalized to a 40 nM fluorescein solution. Error bars indicate standard deviations from two independent measurements performed on different days.

For IP-10

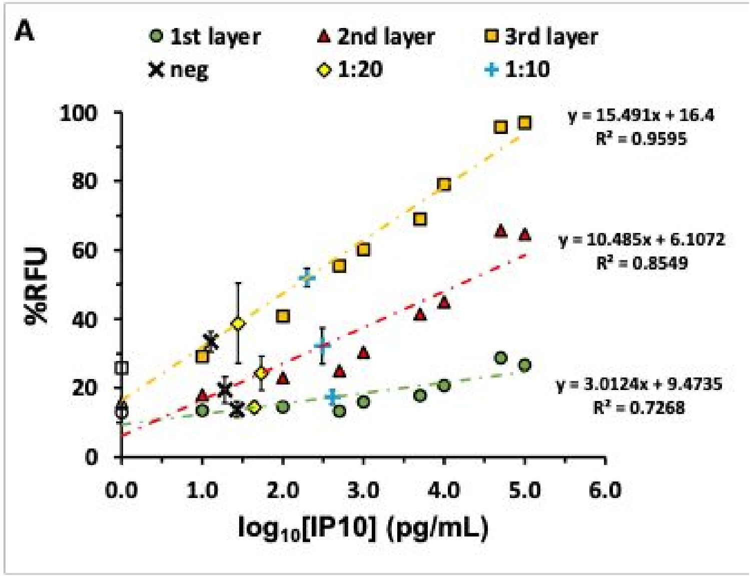

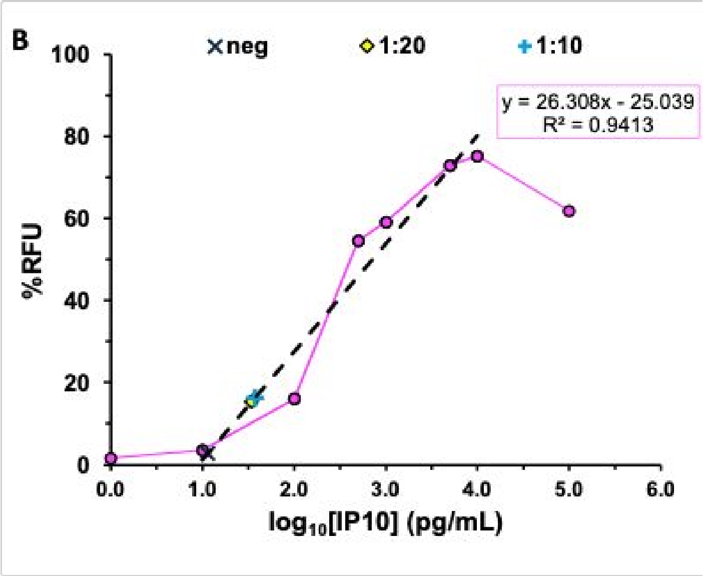

Figure S7 Comparison of the Multilayered Fluorescent Assay and Standard ELISA for detecting IP-10 concentrations in mouse serum samples collected five days post-infection with the DENV2 virus and from mock (saline-treated) controls. (A) Detection using the Multilayered Fluorescent Assay. (B) Detection using the Standard ELISA with an ultrared fluorescent substrate. The infected samples were diluted 10-fold or 20-fold. The y-axis (%RFU) represents the fluorescence intensity of the sample normalized to a 40 nM fluorescein solution. Error bars indicate standard deviations from two independent measurements performed on different days.

For MCP-1

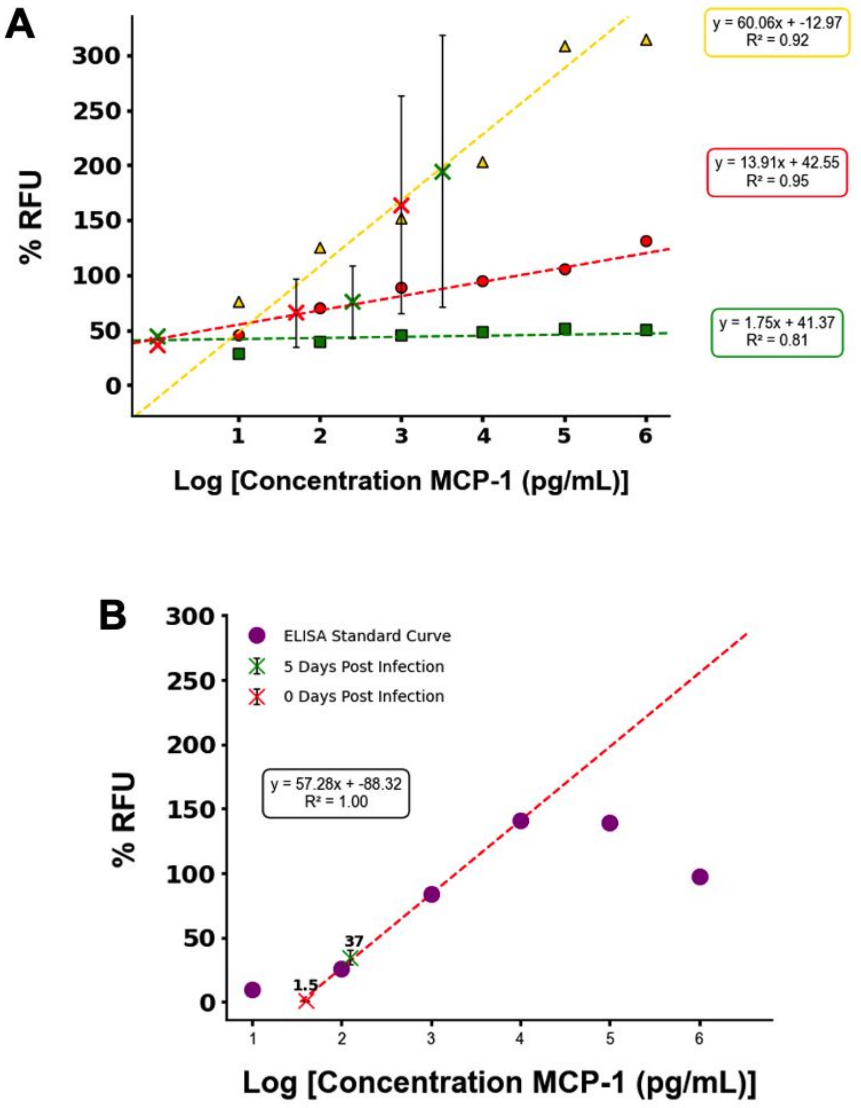

Figure S8 Comparison of the Multilayered Fluorescent Assay and Standard ELISA for detecting MCP-1 concentrations in mouse serum samples collected five days post-infection with the DENV2 virus and from mock (PBS) controls. (A) Detection using the Multilayered Fluorescent Assay. (B) Detection using the Standard ELISA with an ultrared fluorescent substrate. The infected samples were diluted 20-fold. The y-axis (%RFU) represents the fluorescence intensity of the sample normalized to a 100 nM fluorescein solution. Error bars indicate standard deviations from two independent measurements performed on different days.

4. Reproducibility

Intra-assay precision was evaluated by running 24 samples with IP-10 feasibility test (in PBS buffer) in duplicate on the same plate, yielding an average CV (coefficients of variation) of 2.15%.

Table S3 Intra-assay coefficient of variation (CV) for IP-10 detection in duplicate on the same plate (n=24). The RFU% represents the fluorescence intensity of the sample divided by fluorescein standard solution for the biomarkers.

Samples	Run 1 (RFU%)	Run 2 (RFU%)	Mean (RFU%)	Standard deviation (RFU%)	CV (%)
1	7.1	7.7	7.4	0.4	5.34
2	42.9	43.6	43.3	0.5	1.11
3	6.7	6.6	6.6	0.1	0.88
4	33.3	33.6	33.5	0.2	0.70
5	7.5	7.2	7.3	0.2	2.75
6	31.6	31.9	31.7	0.2	0.53
7	8.5	8.1	8.3	0.3	3.32
8	27.3	27.7	27.5	0.3	0.93
9	5.6	5.2	5.4	0.2	4.60
10	46.2	46.0	46.1	0.2	0.34
11	6.0	5.4	5.7	0.4	7.48
12	33.8	33.3	33.6	0.3	0.98
13	7.8	7.3	7.6	0.3	4.24
14	33.1	32.7	32.9	0.2	0.68
15	8.0	7.4	7.7	0.4	5.55
16	33.1	32.7	32.9	0.2	0.68
17	15.2	14.4	14.8	0.5	3.64
18	78.0	78.2	78.1	0.1	0.16
19	12.9	12.3	12.6	0.4	3.10
20	66.9	66.9	66.9	0.0	0.07
21	16.4	16.0	16.2	0.3	1.58
22	75.1	74.8	75.0	0.2	0.25
23	13.8	13.4	13.6	0.3	2.36
24	66.9	66.7	66.8	0.1	0.20

To evaluate inter-assay variability, taking IP-10 as an example, eight concentrations (0 to 100,000 pg/mL) of IP-10 spiked in serum were tested across three independent days. The overall average inter-assay coefficient of variation (CV) across all concentrations and amplification layers was 12.24%, with individual CVs ranging from 2.48% to 31.45%.

Table S4 Inter-assay coefficient of variation (CV) for IP-10 detection across three independent days (n=8). The RFU% represents the fluorescence intensity of the sample divided by fluorescein standard solution for the biomarkers.

	**[C] (pg/mL)**	**Plate 1 (RFU%)**	**Plate 2 (RFU%)**	**Plate 3 (RFU%)**	**Mean (RFU%)**	**Standard deviation (RFU%)**	**CV (%)**
**First layer**	0	10.3	7.6	8.4	8.8	1.4	15.97
	0.1	10.2	8.9	9.5	9.6	0.6	6.76
	1	12.4	10.1	8.9	10.5	1.8	17.29
	10	16.0	11.0	8.7	11.9	3.7	31.45
	100	17.7	17.5	13.4	16.2	2.4	15.03
	1000	20.2	22.8	17.6	20.2	2.6	12.85
	10000	23.5	23.5	19.9	22.3	2.1	9.32
	100000	26.8	32.6	17.8	25.7	7.5	29.01
**Second layer**	0	11.8	12.0	9.6	11.1	1.3	11.98
	0.1	10.7	13.1	10.9	11.6	1.3	11.38
	1	14.3	15.0	12.4	13.9	1.3	9.48
	10	18.9	21.2	15.7	18.6	2.7	14.77
	100	28.4	27.8	26.8	27.7	0.8	3.01
	1000	34.9	34.8	29.9	33.2	2.8	8.56
	10000	36.7	36.7	30.2	34.5	3.7	10.80
	100000	50.9	43.7	32.7	42.4	9.2	21.69
**Third layer**	0	16.2	16.8	17.0	16.7	0.4	2.48
	0.1	18.2	16.2	17.5	17.3	1.0	5.92
	1	20.5	19.5	16.6	18.8	2.0	10.79
	10	26.7	30.3	25.6	27.6	2.5	8.93
	100	34.8	38.4	34.9	36.0	2.1	5.74
	1000	39.7	43.3	40.2	41.1	1.9	4.72
	10000	50.0	64.2	50.5	54.9	8.0	14.63
	100000	68.5	80.9	65.7	71.7	8.1	11.26

Notably, the mean CVs for the first, second, and third layers were 17.21%, 11.46%, and 8.06%, respectively. Reducing CV with increasing layers was observed for NS1 as well, indicating improved precision with additional signal amplification. These results confirm the assay's reproducibility across runs and highlight its robustness for detecting biomarkers within clinically relevant ranges.

Table S5 Summary of inter-assay coefficient of variation (CV) values for NS1 and IP-10 detection in serum across three amplification layers. Inter-assay CVs were calculated based on measurements collected across three independent assay days. Results show decreasing CVs from the first to third layer for both biomarkers, indicating improved precision with signal amplification. Each value represents the average CV across multiple concentrations (NS1: n = 7; IP-10: n = 8), with the overall column representing the mean CV across all layers.

Biomarkers	First layer (%)	Second layer (%)	Third layer (%)	Overall (%)
**NS1 (n=7)**	18.78	12.05	9.83	13.55
**IP-10 (n=8)**	17.21	11.46	8.06	12.24
